# Phytochemicals From Herbs and Spices: Their Absorption, Metabolism, and Relationship to Clinical Outcomes

**DOI:** 10.1093/nutrit/nuag022

**Published:** 2026-05-26

**Authors:** Yudai Huang, Kristina S Petersen, Penny M Kris-Etherton, Indika Edirisinghe, Amandeep K Sandhu, Britt M Burton-Freeman

**Affiliations:** Department of Food Science and Nutrition and Center for Nutrition Research, Institute for Food Safety and Health, Illinois Institute of Technology, Chicago, IL 60616, United States; Department of Nutritional Sciences, Pennsylvania State University, University Park, PA 16802, United States; Department of Nutritional Sciences, Pennsylvania State University, University Park, PA 16802, United States; Department of Food Science and Nutrition and Center for Nutrition Research, Institute for Food Safety and Health, Illinois Institute of Technology, Chicago, IL 60616, United States; Department of Food Science and Nutrition and Center for Nutrition Research, Institute for Food Safety and Health, Illinois Institute of Technology, Chicago, IL 60616, United States; Department of Food Science and Nutrition and Center for Nutrition Research, Institute for Food Safety and Health, Illinois Institute of Technology, Chicago, IL 60616, United States

**Keywords:** herbs and spices, phytochemicals, metabolites, pharmacokinetics, clinical nutrition

## Abstract

This article appears as part of the supplement “The Role of Spices and Herbs on Supporting Healthy Diets and Improving Nutritional Status,” sponsored by the McCormick Science Institute. Results of clinical trials suggest that herbs and spices (H/S) may provide health benefits by influencing risk factors associated with chronic diseases. Phytochemicals found in H/S, such as terpenoids, flavonoids, and phenolic acids, are biologically active compounds that are potentially responsible for these beneficial effects. In this review, we have summarized our past and current research on the phytochemical composition of H/S and the metabolic fate of these compounds following both acute and regular intake of selected H/S. We also briefly discuss associations with clinical outcomes. Our data indicate that certain phytochemicals unique to specific H/S, such as piperine in black pepper and carnosic acid in rosemary, are metabolized after intake and can remain in the circulation for extended periods. In contrast, others are cleared more rapidly, often within a few hours. Some phytochemical metabolites that are cleared more slowly in the bloodstream may serve as biomarkers of H/S intake. Postprandial evaluation and kinetic profiling offer valuable insights into the absorption and clearance patterns of these compounds, shedding light on the time frames during which they may exert biological effects. Tracking metabolite kinetics alongside changes in clinical markers helps to clarify the potential contribution of specific phytochemicals to health outcomes. Further research is needed to explore dose-dependent effects of H/S on circulating metabolites and clinical outcomes, which will enhance our understanding of the mechanisms that account for their biological activity.

## INTRODUCTION

Herbs and spices (H/S) have been utilized as folk medicine in different parts of the world for centuries and have significance in cultural, religious and traditional healing practices. In recent years, research has focused on the health benefits of H/S, including the mechanism(s) and constituent(s) responsible for their biological effects. As seasoning and flavoring agents, H/S can replace excess salt, sugar, and saturated fat in recipes to align with dietary recommendations.^1^^,^^2^ H/S also contain an array of biologically active components, such as flavonoids, phenolic acids and terpenoids, which have antioxidant, antiinflammatory, anticancer, and antidiabetic properties as demonstrated in various animal and human studies.^3–5^ Upon Ingestion, H/S are metabolized by both host and microbial enzymes expanding the pool of bioavailable compounds/metabolites that influence biological processes.^6^ In this article, we summarize our past and recent findings on the phytochemical composition of H/S and the metabolic fate of H/S when consumed by humans after short- and longer-term intake. The relationship with clinical endpoints measured concomitantly in our laboratory is also summarized.

## CHARACTERIZATION OF H/S PHYTOCHEMICALS USED IN CLINICAL TRIALS

Phytochemicals are secondary plant metabolites generated for various purposes, including defense and protection against biotic and abiotic stressors, as well as attracting birds and bees for pollination and seed dispersal.^1^^,^^7^ Accordingly, different plants have distinct types and varying concentrations of phytochemicals. Connecting intake of these with health outcomes requires characterization of the phytochemicals in foods and ingredients.

In our previous clinical trial investigating the effect of acute intake of H/S on cardiometabolic risk factors, we used McCormick brand H/S (Italian herb mixture: thyme, rosemary, basil, oregano, parsley in 1:1 ratio; cinnamon; pepper; and pumpkin pie spice mixture: cinnamon, ginger, nutmeg, allspice, ratio unknown) incorporated into a traditional Taiwanese pancake at a ratio of 1 g per approximately 135 kcal.^6^^,^^8^ A total of 79 phytochemicals from the H/S mixtures were identified and quantified on ultra–high-performance liquid chromatography coupled with triple quadrupole mass spectrometer (UHPLC–QQQ-MS), including 36 flavonoids, 8 terpenoids, 27 phenolic acids, and 9 other compounds. Identification was based on retention time and fragmentation patterns compared with reference standards, databases, and published literature. Some of these phytochemicals are unique to specific H/S, for example, piperine is exclusively found in black pepper, while cinnamaldehyde and coumarin are only found in cinnamon. Alternatively, other phytochemicals are found in different amounts in a variety of H/S, such as hydroxycinnamic acids and hydroxybenzoic acids, presenting in both the Italian herb mixture and cinnamon. In 100 g of the Italian herb mixture, cinnamon, and pumpkin pie spice mixture, the contents of total flavonoids were 763 ± 46, 981 ± 59, and 655.8 ± 39 mg respectively, while the amounts of total phenolic acids were 879.0 ± 53, 11.2 ± 0.7, and 17.1 ± 1 mg per 100 g, respectively. Flavonoids in the Italian herb mixture included flavonols, flavonone, and flavones. Flavonoids in cinnamon and pumpkin pie spice consisted of condensed tannins, including procyanidin types A and B. Phenolic acids included benzoic acid, cinnamic acids, phenylacetic acids, and their derivatives in H/S. Terpenoids were only found in the Italian herb mixture, with a concentration of 498.6 ± 29.9 mg per 100 g.

In addition to the H/S used in this acute study, we also selected 17 H/S for phytochemical analysis used in a separate chronic feeding study conducted at Pennsylvania State University. The selection criteria was to include a broader range of H/S with diverse phytochemical profiles and practical culinary application in recipes, focusing on those consumed at an average of at least 0.1 g per day.^9^^,^^10^ The selected H/S were from 7 botanical families and 6 different parts of the plant (Table 1).

**Table 1.** Selected Herbs and Spices and Major/Unique Phytochemicals^a^^,9,20^

H/S commercial name	Botanical name	Botanic family	Part of the plant	**Major/unique** ^a^ **phytochemicals**	Phenolic acids (mg/100 g), mean ± SD	Flavonoids (mg/100 g), mean ± SD	Other (mg/100 g), mean ± SD
Bay leaf	*Laurus nobilis*	*Lauraceae*	Leaf	Epicatechin, hesperidin	12 ± 0.3	145 ± 14	ND
Basil	*Ocimum basilicum*	*Lamiaceae*	Leaf	Rosmarinic acid, hesperidin	268 ± 7	95 ± 1	ND
Black pepper	*Piper nigrum*	*Piperaceae*	Fruit	Piperine^a^	2 ± 0.2	ND	Piperine: 1620 ± 47
Chili powder	*Capsicum annuum*	*Solanaceae*	Fruit	Rosmarinic acid, capsaicin^a^, dihydrocapsaicin^a^	80 ± 1	64 ± 0.9	Capsaicins: 2.7 ± 0.2
Cilantro	*Coriandrum sativum*	*Apiaceae*	Leaf	Hesperidin	18 ± 2	233 ± 18	ND
Cinnamon	*Cinnamomum verum*	*Lauraceae*	Bark	Cinnamaldehyde^a^, coumarin^a^, proanthocyanins	11 ± 0.3	1 ± 0.1	Coumarin: 685 ± 56
Coriander	*Coriandrum sativum*	*Apiaceae*	Seed	apigenin-7-*O*-glucoside	111 ± 28	7 ± 2	ND
Cumin	*Cuminum cyminum*	*Apiaceae*	Seed	Apigenin-7-*O*-glucoside, luteolin-7-O-glucoside	44 ± 1	262 ± 7	ND
Garlic	*Allium sativum*	*Amaryllidaceae*	Bulb	*S*-allycysteine^a^, gamma-glutamyl-*S*-methylcysteine^a^, methiin^a^, alliin^a^	2 ± 0.2	ND	Sulfur-containing compounds: 133 ± 14
Ginger	*Zingiber officinale*	*Zingiberaceae*	Root	Gingerols^a^, shogaols^a^	2 ± 0.4	ND	Gingerols and shogaols: 229 ± 58
Onion powder	*Allium cepa*	*Amaryllidaceae*	Bulb	*S*-allycysteine^a^, gamma-glutamyl-*S*-methylcysteine^a^, methiin^a^, alliin^a^	0.6 ± 0.04	ND	Sulfur-containing compounds: 51 ± 2
Oregano	*Origanum vulgare*	*Lamiaceae*	Leaf	Rosmarinic acid, apigenin-7-*O*-glucuronide, luteolin-7-O-glucoside	261 ± 2	224 ± 3	ND
Paprika	*Capsicum annuum*	*Solanaceae*	Fruit	Vanillic acid, ferulic acid	17 ± 0.9	4 ± 0.6	ND
Parsley	*Petroselinum crispum*	*Apiaceae*	Leaf	Apigenin-7-O-glucoside	17 ± 0.6	28 ± 2	ND
Red pepper	*Capsicum annuum*	*Solanaceae*	Fruit	Capsaicin^a^, dihydrocapsaicin^a^	16 ± 2	0.2 ± 0	Capsaicins: 99 ± 3
Rosemary	*Salvia rosmarinus*	*Lamiaceae*	Leaf	12-Methoxycarnosic acid^a^, carnosic^a^ acid, rosmanol^a^, rosmarinic acid	616 ± 57	136 ± 20	Terpenoids: 1299 ± 185
Turmeric	*Curcuma longa*	*Zingiberaceae*	Root	Curcuminoids^a^	25 ± 1	ND	Curcuminoids: 3649 ± 385

Abbreviation: ND, not detected.

a Unique to specific herbs or spices.

Untargeted analyses for classification and clustering involved scanning ions with a mass to charge ratio (m/z) of 100-3000 from extracted H/S samples on an UHPLC system equipped with a quadrupole time-of-flight (Q-TOF) mass spectrometer.^9^ Collected data were extracted on Agilent MassHunter Profinder and imported into Agilent Mass Profiler Professional software. Multivariate statistical analyses were performed on the subsets of entities after filtering of a total of 437 unidentified entity groups from the extracted data, and indicated the similarity based on the detected entity groups in *Solanaceae, Lauraceae*, or *Amaryllidaceae* families. We also observed that phytochemical compositions can vary significantly even when H/S originate from the same plant or belong to the same botanical family. This study indicated a potential to classify H/S based on their phytochemical profiles and suggests that compared to using a single herb or spice at the same dosage, using mixtures of H/S may provide enhanced health benefits due to their diverse phytochemical composition.

Targeted analyses using multiple reaction monitoring transitions on a UHPLC-QQQ-MS system revealed a total of 67 phytochemicals from the selected H/S. Certain unique phytochemicals are responsible for the pungent or tangy flavor of H/S. For example, sulfur-containing compounds (*S*-allycysteine, methiin, and alliin) are only found in garlic and onion powder; gingerols and shogaols are unique to ginger; and capsaicin is usually found in *Capsicum annum* spices (eg, chili powder and red pepper) responsible for the spicy or hot flavor. Curcuminoids are natural pigment compounds found in turmeric providing its characteristic yellow color. The unique phytochemicals present in the specific H/S may be used as biomarkers of intake after consumption. Concentrations of total phenolic acids, flavonoids, and other signature compounds of each H/S are shown in Table 1.

## METABOLIC FATE OF H/S PHYTOCHEMICALS

### Metabolites Profile over 24 hours after Intake

**Figure 1.**
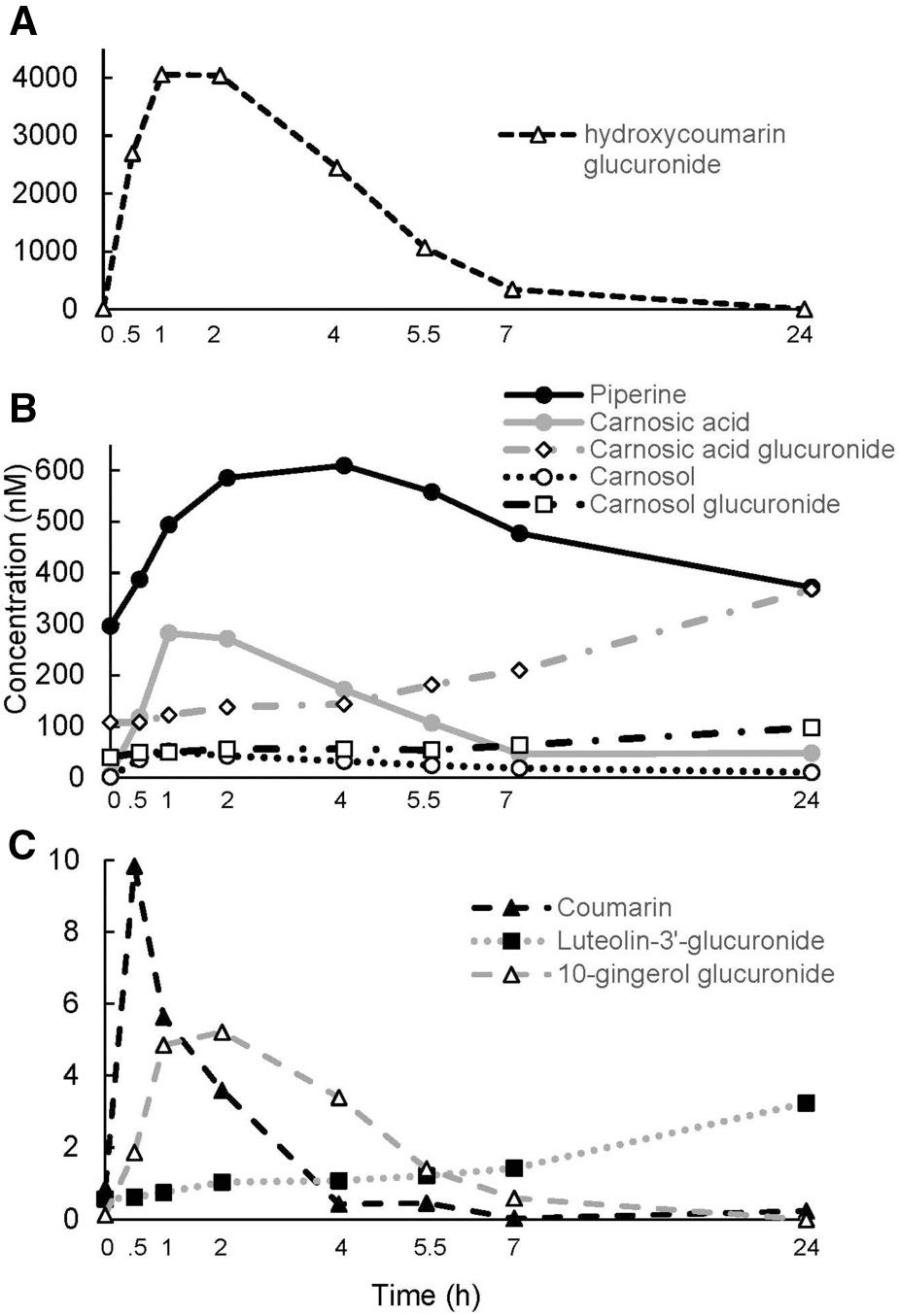
Kinetic profile (0-24 hours) of potential biomarker metabolites. Compound peak concentrations: (A) higher than 4000 nM (hydroxycoumarin glucuronide generated after consuming cinnamon); (B) 100-600 nM (piperine generated after consuming black pepper; carnosic acid, carnosol, and their conjugated glucuronides generated after consuming the Italian herbs); (C) below 10 nM after acute intake of herbs and spices (H/S) mixtures (coumarin generated after consuming cinnamon; luteolin-3′-*O*-glucuronide generated after consuming the Italian herbs; 10-gingerol glucuronide generated after consuming pumpkin pie spice)^6^

**Figure 2.**
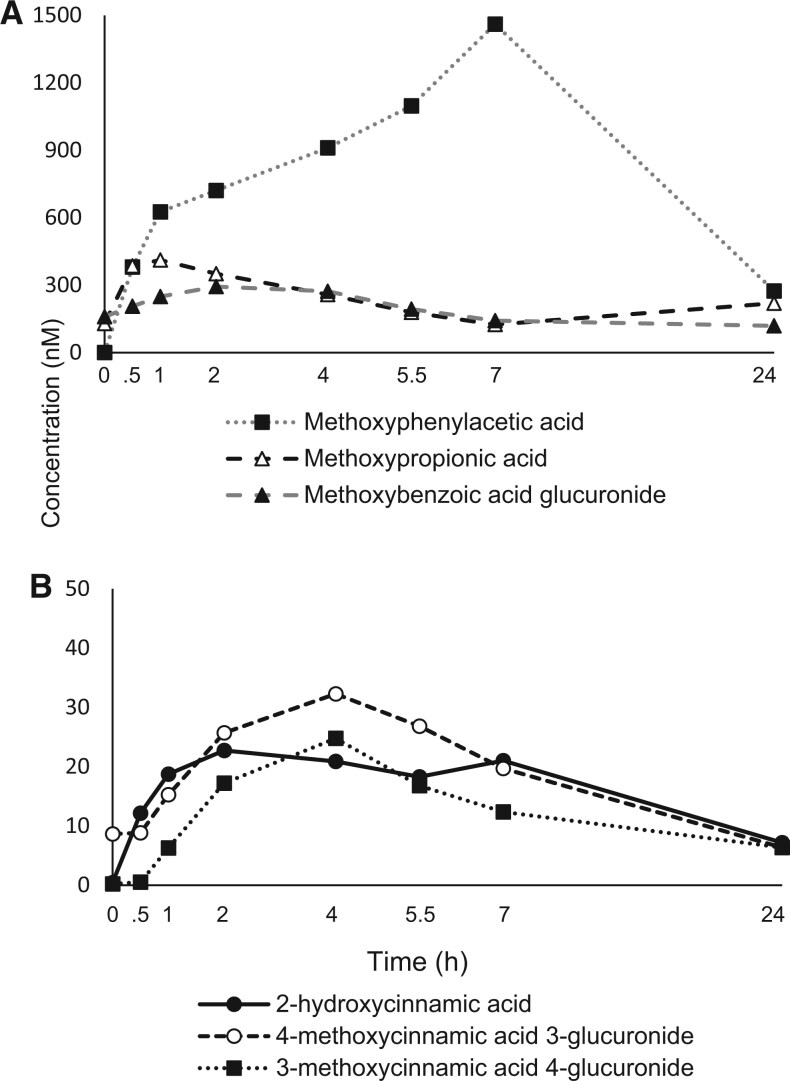
Kinetic profile (0-24 hours) of phenolic acid metabolites. Compound peak concentrations (A) between 100-1500 nM; (B) below 40 nM after acute intake of herbs and spices (H/S) mixtures. Data are shown as the average after consuming all test meals, except 2-hydroxycinnamic acid and methoxyphenylacetic acid increased after consuming meals containing cinnamon^6^

Limited research is available on the metabolic fate and pharmacokinetic (PK) profile of phytochemicals from H/S after consumption in humans. We analyzed postprandial plasma samples from a randomized, single-blinded, 4-arm, 24-hour, crossover clinical trial conducted in individuals (*n* = 24), aged 37 ± 3 years with a body mass index of 28.4 ± 0.6 kg/m^2^.^6^ Blood samples were collected at 0, 0.5, 1, 2, 4, 5.5, 7, and 24 hours after study participants consumed a Taiwanese pancake breakfast with only pepper and salt (control), or after the control meal plus 1 of the 3 H/S mixtures (Italian herb, cinnamon or pumpkin pie spice). The Taiwanese pancake breakfast was based on each individual’s calorie requirements to maintain body weight. Breakfast provided 35% of an individual’s daily caloric intake with H/S added at a rate of 1 g per approximately 135 kcal.^6^^,^^8^ The plasma samples at different time points were analyzed using UHPLC-QTOF-MS and UHPLC-QQQ-MS, respectively. A total of 47 metabolites were identified and quantified. The key findings were that phytochemicals identified in H/S were bioavailable, appearing in blood either as parent compounds and/or transformed compounds (ie, metabolites) peaking and clearing on different time scales. For example, piperine, the signature compound of black pepper, increased after ingestion of all breakfast meals, peaking in the blood 3.4-5.0 hours after intake. Coumarin and hydroxycoumarin glucuronide increased only after ingestion of the pancake made with cinnamon or the pancake made with the pumpkin spice mixture, which contains cinnamon, peaking 2 hours after intake. Carnosic acid, carnosol, and their conjugated glucuronides increased only after ingestion of the pancake made with the Italian herb mixture, showing the maximum concentrations in blood at 24 hours. Unlike the aforementioned results, phenolic acid metabolites may be less specific to the H/S consumed. For example, methoxyphenyl acetic acids, hydroxybenzoic acids, and hydroxycinnamic acids increased markedly after intake of all of the H/S incorporated into pancakes. Metabolites appearing in the blood after 4 hours of intake are generally degradation products of parent compounds or are generated by gut microbiota, during transitions in the gastrointestinal tract. Figures  1 and 2 show plasma concentrations of metabolites peaking and returning to baseline at different time points over a 24-hour interval based on our previously published study.^6^ These data provide valuable insights regarding the relationship between H/S intake and clinical outcomes acutely and moreover guide hypothesis testing and study design (ie, dosing) when the long-term effects of H/S intake are investigated.

### Metabolites Profile after 4 Weeks H/S Intake

To understand phytochemical absorption and metabolism patterns after regular intake and dose response, we analyzed blood samples from a randomized, 3-period crossover, controlled-feeding clinical trial.^10^^,^^11^ Participants (*n* = 63) consumed standardized diets with 0.5, 3.3, or 6.6 g/d/2100 kcal H/S daily for 4 weeks with washout periods of least 2 weeks between dosage levels. Fasting blood samples were initially taken from the group of participants who received the highest dose (6.6 g H/S daily) and pooled together to obtain a representative sample (*n* = 10). In the representative blood sample a total of 91 metabolites were characterized and quantified on UHPLC-QQQ-MS, including 71 phenolic acids (3894 ±67 nM), 11 terpenoids (545 ± 48 nM), 3 flavonoids (6 ±0.7 nM), 3 sulfur-containing compounds (496 ± 110 nM), and 3 other compounds (0.23 ± 0.05 nM capsaicin glucuronide isomer, 68 ± 4 nM hydroxycoumarin isomer, and 41 ± 9 nM piperine). We further confirmed the presence of conjugated glucuronide curcuminoids, gingerols, and capsaicin in the blood samples on UHPLC-QTOF-MS by comparing the observed mass to charge ratio (m/z) and the fragmentation patterns with those reported in the published literature and databases. The detection of specific metabolites in blood samples supports the role of parent phytochemicals in H/S as intake biomarkers, such as sulfur-containing compounds for onion and garlic and capsaicin for red pepper and chili powder. Quantification of phytochemical metabolites in fasting blood from all dosage levels is ongoing.

### Comparison of Metabolites after Acute and Longer-Term Intake

Herbs and spices (H/S) contain a diverse array of phytochemicals. The bioaccessibility, bioavailability, and metabolic handling of these phytochemicals differ depending on their structure and the food matrices in which they are incorporated. Basil, black pepper, cinnamon, ginger, oregano, parsley, and rosemary were used in both the acute and chronic study meals, but at different concentrations. Likewise, some metabolites were detected both in postprandial blood samples after acute intake and in fasting blood samples after regular intake. For example, piperine increased approximately 2-fold to a maximum concentration between 3.4-5 hours after a single intake of 0.6 g black pepper in a Taiwanese pancake meal, but was also quantified, albeit in much lower concentrations, in fasting pooled blood after an average intake of 0.5 g black pepper daily for 4 weeks in diet-controlled conditions. Piperine is lipophilic in nature and subject to enterohepatic recirculation which may explain, at least in part, these observations.^12^ Piperine is also a well-known bioenhancer for select drugs and other bioactive compounds.^13^ Piperine can alter PK profiles of other compounds by inhibiting the efflux transporter P-glycoprotein and enzyme CYP3A4, both of which are involved in xenobiotic elimination.^13^ Additionally, piperine may inhibit the enzyme uridine 5′-diphospho-glucuronosyltransferase and therefore absorbed compounds remain unconjugated after absorption.^14^ It is possible that some compounds in our study may have greater bioavailability, altered absorption and conjugation kinetics, and possibly slower clearance due to their co-administration with black pepper in the study meal. However, tracer studies would be required to fully elucidate the differences.

Once these phytochemicals are absorbed, they can undergo both phase I (ie, hydroxylation) and phase II metabolism (ie, glucuronidation, methylation, sulfation). The major phase II metabolites observed in our previous study^6^ were glucuronide-conjugated compounds with varied peak and clearance curves. Carnosic acid glucuronide and carnosol glucuronide concentrations were still increasing at 24 hours after consumption of a Taiwanese pancake containing 6 g Italian herb mixture (24-hour concentrations: 369.8 ± 61.1 and 98.5 ± 18.3 nM, respectively), whereas concentrations of the unconjugated parent compound peaked 1-2 hours after ingestion.^6^ The same glucuronidated metabolites were also elevated after eating meals containing only 0.2 g rosemary for 4 weeks (170 ± 16 nM carnosic acid glucuronide; 176 ± 11 nM carnosol glucuronide). In contrast, coumarin peaked sharply at approximately 0.5 hours (10.4 ± 2.1 nM) and hydroxycoumarin glucuronide (phase I/phase II metabolite) peaked at 1-2 hours at several times higher concentrations of the unconjugated intact form (4374 ± 487 nM); both were eliminated by 24 hours after intake of 6 g cinnamon in the Taiwanese pancake meal. Correspondingly, neither coumarin nor its conjugate metabolites were detected in the fasting pooled blood samples after daily intake of 1.2 g cinnamon for 4 weeks. The nonpolar structure of coumarin allows for passive diffusion and quick appearance in circulation,^16^ but then its extensive metabolism and half-life (<2 hours) results in rapid clearance.^17^ Many other phytochemicals, such as flavonoids, have sugar moiety attached (for example, luteolin-7-*O*-glucoside, apigenin-7-*O*-glucoside, and hesperidin, Table 1) that influence absorption profiles. Prior to absorption, these phytochemicals generally require hydrolysis of glycosidic units by host or microbial enzymes, such as by lactase-phlorizin hydrolase or cytosolic β-glucosidase.^18^^,^^19^ Not all glycosylated flavonoids are absorbed in the same way. Factors such as types of sugar moiety, positions of glycosidic bonds, and structure of the flavonoids can affect the absorption routes.^19^

The PK profiles of H/S phytochemical metabolites in postprandial blood samples after a single intake may help to explain the presence or absence of specific metabolites in fasting blood samples after regular intake. Additionally, various smaller phenolic acids, including derivatives from benzoic acids, cinnamic acids, hippuric acids, phenylacetic acids, and phenylpropionic acids were detected in blood samples from both studies. These phenolic acids can be products of flavonoid and amino acid catabolism or products of microbial metabolism, indicating the role of gut microbiota in generating potentially bioactive metabolites after H/S intake.

## H/S PHYTOCHEMICAL METABOLITES AND CLINICAL OUTCOMES

Prior research had indicated a potential benefit of H/S intake on measures of vascular function, specifically flow-mediated dilation (FMD).^8^^,^^20^ Other investigators have reported acute benefits on metabolic endpoints,^20–22^ inflammation,^23^^,^^24^ and oxidative stress endpoints.^25^^,^^26^ To better understand the metabolites potentially involved in these actions, we measured similar vascular and metabolic endpoints in our acute H/S study discussed above.^8^ In this study, FMD significantly increased at 24 hours after intake of the Taiwanese pancake breakfast meals containing Italian herbs (*P* = .048) and pumpkin pie spice (*P* = .027) compared to the control meal.^8^ Metabolites significantly elevated at this time included conjugated glucuronides of carnosic acid, carnosol, and luteolin (Figure 1B and C) suggesting a potential role of these compounds in mechanisms underlying endothelial function. We also reported attenuation of the glucose response after the breakfast meal containing cinnamon compared to the control (meal × time interaction, *P* = .003). This effect may have been related to the significant elevation of hydroxycoumarin glucuronide increasing approximately 360-fold from baseline, reaching 4374 ± 487 nM after consumption of the Taiwanese pancake made with 6 g cinnamon (Figure 1A). We also reported improvement in insulin responses after eating the H/S meals compared to a control (no H/S) meal, particularly with cinnamon, but improvements were also evident after eating breakfast meals containing the Italian herb and pumpkin spice mix, wherein effects were modified by a significant age effect (meal × time × age, *P* = .001). In people older than 40 years, less insulin was required to manage postmeal glucose compared to the individuals who were younger than 40 years. The small and uneven sample size and variance precluded making conclusions, but these data provide insights into future directions for understanding dose-exposure-age interrelationships. Additionally, in the 4-weeks regular intake study, daily intake of 6.6 g/d/2100 kcal H/S lowered mean 24-hour systolic blood pressure compared with dosage levels at 3.3 or 0.5 g/d/2100 kcal.^10^ From the acute data, candidate metabolites for influencing blood pressure include glucuronide-conjugated carnosic acid, carnosol, luteolin, and apigenin. However, chronic consumption influences various host and microbial enzymatic pathways, which may reveal alternative candidate metabolites responsible for the biological effects. These analyses are ongoing and will shed additional light on H/S PK-pharmacodynamic relationships.

## SUMMARY AND CONCLUSIONS

Complex and distinct phytochemicals in H/S provide flavor, aroma, color, and importantly, health benefits. Some H/S from the same botanical family shared a similar phytochemical profile (*Solanaceae, Lauraceae*, or *Amaryllidaceae*), while other H/S have very different and unique compounds (gingerols in ginger, curcuminoids in turmeric, piperine in black pepper, carnosic acid in rosemary). People often consume H/S as mixtures consisting of diverse varieties of H/S. For example, Italian herb mixture and pumpkin pie mixture contain 5-6 different H/S. These mixtures deliver a broad spectrum of concentrated health-promoting phytochemicals. Our research suggests that these phytochemicals are bioavailable and bioactive after intake and are metabolized by both host enzymes and gut microbiota, which together generate various bioactive metabolites that can be detected after both single and long-term intake. Additionally, these metabolites are associated with improvements in clinical outcomes, such as vascular function and hyperinsulinemia management. Our findings reinforce the therapeutic value of including H/S into the daily diet–a concept supported by traditional folklore medicine and aligned with modern day “Food is Medicine” approaches.
